# Mg-BGNs/DCECM Composite Scaffold for Cartilage Regeneration: A Preliminary In Vitro Study

**DOI:** 10.3390/pharmaceutics13101550

**Published:** 2021-09-24

**Authors:** Zhiguo Yuan, Zhuocheng Lyu, Xin Liu, Jue Zhang, You Wang

**Affiliations:** 1 Department of Bone and Joint Surgery, Renji Hospital, School of Medicine, Shanghai Jiaotong University, Shanghai 200127, China; yzgad@163.com (Z.Y.); lzc2015@sjtu.edu.cn (Z.L.); lxcola@sjtu.edu.cn (X.L.); 2 School of Stomatology, Wannan Medical College, Wuhu 241002, China

**Keywords:** bioactive glasses (BGs), decellularized cartilage extracellular matrix (DCECM), osteochondral lesions, tissue engineering, composite scaffold

## Abstract

Cartilage lesions can lead to progressive cartilage degeneration; moreover, they involve the subchondral bone, resulting in osteoarthritis (OA) onset and progression. Bioactive glasses, with the dual function of supporting both bone and cartilage regeneration, have become a promising biomaterial for cartilage/bone engineering applications. This is especially true for those containing therapeutic ions, which act as ion delivery systems and may further promote cartilage repair. In this study, we successfully fabricated Mg-containing bioactive glass nanospheres (Mg-BGNs) and constructed three different scaffolds, DCECM, Mg-BGNs-1/DCECM (1% Mg-BGNs), and Mg-BGNs-2/DCECM (10% Mg-BGNs) scaffold, by incorporating Mg-BGNs into decellularized cartilage extracellular matrix (DCECM). All three scaffolds showed favorable microarchitectural and ion controlled-release properties within the ideal range of pore size for tissue engineering applications. Furthermore, all scaffolds showed excellent biocompatibility and no signs of toxicity. Most importantly, the addition of Mg-BGNs to the DCECM scaffolds significantly promoted cell proliferation and enhanced chondrogenic differentiation induction of mesenchymal stem cells (MSCs) in pellet culture in a dose-dependent manner. Collectively, the multifunctional Mg-BGNs/DCECM composite scaffold not only demonstrated biocompatibility but also a significant chondrogenic response. Our study suggests that the Mg-BGNs/DCECM composite scaffold would be a promising tissue engineering tool for osteochondral lesions, with the ability to simultaneously stimulate articular cartilage and subchondral bone regeneration.

## 1. Introduction

It is well known that articular cartilage lesions can lead to progressive cartilage degeneration; moreover, these lesions involve the subchondral bone. This results in an early onset of osteoarthritis (OA) [1,2]. OA is a leading cause of disability among adults [3]. As articular cartilage lacks an innate self-repair ability with avascularity, it poses a significant challenge for cartilage regeneration [4]. Tissue engineering strategies, which involve a combination of cells and bioactive scaffolds, have become a promising approach for cartilage repair and regeneration [5]. Bioactive scaffolds play a significant role in tissue engineering [6]. The ideal scaffold biomaterials should be biocompatible, biodegradable, and have a porous architecture that can promote cell adhesion and proliferation. In particular, the scaffold should provide bioactive stimuli for target tissue regeneration and formation [7].

For cartilage tissue engineering, decellularized cartilage extracellular matrix (DCECM) is a promising scaffold biomaterial. This is due to its ability to provide a cartilage-specific extracellular matrix for cell–matrix interactions, such as collagen II, glycosaminoglycans (GAGs), and certain active growth factors [8,9]. It has been found that the cartilage extracellular matrix can promote chondrogenesis of mesenchymal stem cells (MSCs) and reduce the dedifferentiation phenomenon of chondrocytes during in vitro expansion [10,11]. In our previous studies, we also found that cartilage-derived extracellular matrix scaffolds can promote stem cell migration, proliferation, cartilage differentiation, and enhance in vivo cartilage defect repair; therefore, it can be considered a promising alternative tissue engineering scaffold for the treatment of articular cartilage defects [12,13]. However, this cartilage-derived matrix, though a promising biomaterial for cartilage regeneration, still has some challenges. First, the physicochemical decellularization process may lead to a loss of bioactive factors that are beneficial for cartilage regeneration. Second, some special bioactive stimuli factors (such as therapeutic ions) may be absent in the native cartilage matrix. Furthermore, some articular cartilage lesions often involve subchondral bone injury. Additionally, it is important to simultaneously promote cartilage and subchondral bone regeneration for osteochondral defects in OA [14,15]. Therefore, to enhance the chondrogenic ability of cartilage tissue engineering scaffolds, cartilage-derived matrix scaffolds may be functionalized further.

Many studies have demonstrated that certain therapeutic ions play a significant role in connective tissue development and maintenance, especially in the articular cartilage and bone [16,17]. Mg is a very important element in the maintenance of normal metabolism in the human body. It also plays a key role in skeletal growth and development. Mg deficiency has been reported to lead to cartilage lesions during development [18]. Additionally, Mg ions play a bipolar role in MSC proliferation and chondrogenesis. Low doses of magnesium can promote cell proliferation, whereas high doses can promote cell differentiation [19,20]. Hu et al. found that Mg could enhance the chondrogenic differentiation of MSCs by inhibiting macrophage-induced inflammation [21]. Furthermore, Mg could be incorporated into hydroxyapatite (HA) crystals, and benefit bone regeneration [22,23]. Si is a nutrient element in the human body; it is important for the metabolism and growth of healthy cartilage and bone [18,24]. It has been reported that silicon can promote MSC proliferation and osteogenic differentiation [25]. It is also able to stimulate cartilage extracellular matrix (ECM) synthesis and deposition [26].

Having an adjustable composition and specific glass network structure properties, means bioactive glass is a promising ion carrier vehicle for controlled ion release. Previous studies have demonstrated that ionic dissolution products from bioactive glass are involved in cell proliferation, differentiation, and metabolic activity [27]. Traditionally, owing to their excellent osteogenic properties, bioactive glasses (BGs) have been widely used for bone tissue regeneration. Recently, it has been found that certain therapeutic ion-containing BGs are beneficial for chondrogenic differentiation of MSCs and might be able to further promote cartilage repair [28,29]. As cartilage lesions in OA often involve subchondral bone injury, BGs, having the aforementioned medical capabilities, might be a promising biomaterial for osteochondral regeneration. Many previous studies have reported that Mg-containing bioactive glass could promote osteogenic differentiation of MSCs and could be used for bone regeneration [30]. However, there is relatively little research on the cartilage regeneration effect of Mg-containing bioactive glass.

As previously stated, certain therapeutic ions, such as magnesium and silicon, have positive impacts on the chondrogenic differentiation of MSCs. We hypothesized that Mg-containing bioactive glass would be beneficial for cartilage regeneration. Therefore, in this research, we fabricated the Mg-containing bioactive glass nanospheres (Mg-BGNs) with the Sol-gel method. We then used Mg-BGNs to functionalize the DCECM scaffold to get an optimized cartilage tissue engineering scaffold, Mg-BGNs/DCECM composite scaffold. We observed the physicochemical characteristics, cytocompatibility, and cartilage induction effect of the Mg-BGNs/DCECM scaffold and evaluated the feasibility of the application of Mg-BGNs/DCECM scaffolds for cartilage regeneration in vitro.

## 2. Materials and Methods

### 2.1. Fabrication and Characterization of Scaffolds

#### 2.1.1. Preparation of Mg-BGNs

Mg-containing bioactive glass (Si:Ca:Mg = 80:10:10) nanospheres (Mg-BGNs) were fabricated using the modified Stöber method. In brief, 12 mL of tetraethyl orthosilicate (TEOS, 99%) mixed with 48 mL of ethanol (96% VWR) was added to a solution containing 18 mL of ammonium hydroxide (25.0%), 33 mL of ethanol, and 100 mL of deionized water. The reaction was allowed to proceed for 30 min following which a certain amount of calcium nitrate tetrahydrate (99%) and magnesium nitrate tetrahydrate (99.8%) were added. After stirring for 90 min, the deposits were collected by centrifugation (8000 rpm), washed, dried, and finally calcinated at 700 °C for 4 h. The designed compositions of the Mg-BGNs are 80SiO_2_–10CaO–10MgO in mol. All chemicals were purchased from Sigma-Aldrich and used as received without further purification.

#### 2.1.2. Preparation of Decellularized Cartilage Extracellular Matrix (DCECM)

The DCECM was fabricated using our previous differential centrifugation method [31,32]. The swine articular cartilage was washed with distilled water, cut into pieces (1 mm × 1 mm × 1 mm), and then treated with hydrogen peroxide (Sigma-Aldrich, St. Louis, MO, USA). After washing with distilled water, the minced cartilage was homogenized using a machine (Kinematica AG, Lucerne, Switzerland) at a low temperature (4 °C). The decellularized cartilage matrix was obtained using the differential centrifugation method with a high-speed, low-temperature centrifuge (Thermo, Osterode, Germany). The cartilage homogenate samples were first centrifuged at 2000 rpm for 10 min. Next, the sediment was removed and the supernatant was recentrifuged at 10,000 rpm for 30 min. The supernatant was then removed; the remaining sedimentary slurry was the decellularized cartilage extracellular matrix hydrogel.

#### 2.1.3. Fabrication of DCECM and Mg-BGNs/DCECM Scaffolds

We used a combination of Mg-BGNs and DCECM hydrogel to construct the DCECM and Mg-BGNs/DCECM scaffolds. The DCECM hydrogel was placed into cylindrical molds and then freeze-dried to fabricate the DCECM scaffold. Mg-BGNs (2 mg) were added to 10 mL of decellularized cartilage matrix hydrogel to fabricate the Mg-BGNs-1/DCECM (1% Mg-BGNs-1) composite hydrogel. This was then transferred into cylindrical molds and freeze-dried to construct the Mg-BGNs-1/DCECM scaffold. The Mg-BGNs-2/DCECM (10% Mg-BGNs) composite hydrogel was fabricated by adding 20 mg Mg-BGNs to 10 mL decellularized cartilage matrix hydrogel, which was then transferred into cylindrical molds and freeze-dried to construct the Mg-BGNs-2/DCECM scaffold.

#### 2.1.4. Characterization of Mg-BGNs

The microstructure of Mg-BGNs was observed using scanning electron microscopy (SEM; Gemini 300, Zeiss, Jena, Germany) and transmission electron microscopy (TEM; TALOS F200 X, FEI, Waltham, MA, USA). The constituent elements of the Mg-BGNs were determined using TEM elemental mapping.

#### 2.1.5. Characterization of DCECM and Mg-BGNs/DCECM Scaffolds

The morphology of DCECM, Mg-BGNs-1/DCECM, and Mg-BGNs-2/DCECM scaffolds were observed using a scanning electron microscope (SEM; Gemini 300, Zeiss) after being coated with gold.

The chemical groups of the DCECM, Mg-BGNs-1/DCECM, and Mg-BGNs-2/DCECM scaffolds were identified by Fourier transform infrared spectroscopy (FTIR; Thermo Scientific Nicolet iS5, Waltham, MA, USA). The IR spectra were measured in the range of 2000–400 cm^−1^ at a resolution of 4 cm^−1^.

The chemical constituents and elemental states of the DCECM, Mg-BGNs-1/DCECM and Mg-BGNs-2/DCECM scaffolds were determined using X-ray photoelectron spectroscopy (XPS; Thermo Scientific K-Alpha, Al Kα 1486.6 eV, beam spot size 400 μm, pass energy 100.0 eV, and energy step size 1 eV).

#### 2.1.6. Ion Release of Mg-BGNs/DCECM Scaffolds

The release of Mg, Si, and Ca ions from the Mg-BGNs-1/DCECM and Mg-BGNs-2/DCECM scaffolds was assayed over 7 days using inductively coupled plasma-atomic emission spectroscopy (ICP-AES; Avio 500). Briefly, the Mg-BGNs-1/DCECM and Mg-BGNs-2/DCECM scaffolds (20 mg) were dispersed in 10 mL of phosphate-buffered saline (PBS) in a 37 °C incubator shaker. At the set time points of 3, 6, 12, 24, 48, 96, 120, and 168 h, the samples were collected for ion assessment.

### 2.2. Cytocompatibility of DCECM and Mg-BGNs/DCECM Scaffolds

#### 2.2.1. Isolation and Culture of Rabbit Bone Marrow Mesenchymal Stem Cells (BMSCs)

Rabbit BMSCs were isolated as described in our previous study, and this study was performed according to a protocol approved by the Institutional Animal Care and Use Committee (IACUC) of Renji Hospital affiliated to Shanghai Jiao Tong University Medical College (identification code: RJ2021-0525; date: 20 May 2021). The P0 cells were cultured in α-minimum essential medium (α-MEM; Gibco) with 10% fetal bovine serum (FBS; Gibco) at 37 °C in a 5% CO_2_ incubator. The cells were diluted 1:3 at 90% confluence. Passage 3 was used for cytocompatibility studies.

#### 2.2.2. Cell Seeding

All the DCECM, Mg-BGNs-1/DCECM, and Mg-BGNs-2/DCECM scaffolds were shaped into Ø10 mm × 5 mm cylinders for use. Before seeding the cells, the scaffolds were sterilized and treated with α-MEM (Gibco, Waltham, MA, USA) overnight. Each scaffold was seeded with 5 × 10^5^ BMSCs and the medium was changed (α-MEM with 10% FBS) every 2 days.

#### 2.2.3. Live/Dead Staining and Cell Viability Analysis

The cytotoxicity of the DCECM, Mg-BGNs-1/DCECM, and Mg-BGNs-2/DCECM scaffolds was evaluated using a live/dead assay kit (Beyotime, Shanghai, China). After seeding BMSCs on the DCECM, Mg-BGNs-1/DCECM, and Mg-BGNs-2/DCECM scaffolds and cultured for 1, 3, and 7 days, the cell/scaffold complex was harvested. It was subsequently washed with PBS (Gibco, Life Technologies, Grand Island, NY, USA) and incubated with calcein AM and propidium iodide (PI) for 30 min according to the manufacturer’s instructions (Beyotime, Shanghai, China). After washing with PBS three times, the complex was observed using a confocal microscope (Leica SP8, Germany). Cell viability was calculated as follows: live cells/total cells × 100%. Five horizons per group were used for cell viability analysis, and the images were analyzed using Imaris software (ver. 7.4. software (Bitplane, Zurich, Switzerland).

#### 2.2.4. Cell Proliferation

The proliferation of BMSCs seeded in the scaffolds was evaluated using a CCK-8 assay kit (cell counting kit-8, Beyotime, China). 5 × 10^5^ BMSCs were seeded in the DCECM, Mg-BGNs-1/DCECM, and Mg-BGNs-2/DCECM scaffolds each. After culturing for 1, 3, and 7 days, the cell–scaffold complex was incubated with 10% CCK-8 solution for 4 h at 37 °C in a 5% CO_2_ incubator. The OD values were measured at 450 nm using a multifunction microplate reader (Synergy HT, Bio-Tek Co., Winooski, VT, USA).

#### 2.2.5. Cell Morphology

The morphology of cells seeded on the DCECM, Mg-BGNs-1/DCECM, and Mg-BGNs-2/DCECM scaffolds was observed using a SEM (Gemini 300, Zeiss). The cell/scaffold complex was harvested after culturing for 3 days; it was then fixed with 2.5% glutaraldehyde for 24 h. Subsequently, it was dehydrated with gradient alcohol and dried to a critical point (EM CPD300; Leica, Wetzlar, Germany) using CO_2_. The samples were then sputter-coated with gold. The microstructure of the cell scaffolds was observed using SEM (Gemini 300, Zeiss).

The cytoskeletal architecture of cells seeded on the DCECM, Mg-BGNs-1/DCECM, and Mg-BGNs-2/DCECM scaffolds was visualized by F-actin staining. The cell/scaffold complex was harvested after culturing for 1, 3, and 7 days and fixed with 2.5% glutaraldehyde for 24 h. After co-staining with Fluorescein Isothiocyanate (FITC)-conjugated phalloidin (Solarbio, Beijing, China) and DAPI (Beyotime, Shanghai, China), these samples were observed using a Leica SP8 CLSM.

### 2.3. Chondrogenic Differentiation Induction of DCECM and Mg-BGNs/DCECM Scaffolds

#### 2.3.1. Chondrogenic Differentiation Induction in BMSC Pellets

We evaluated the induction of chondrogenic differentiation of DCECM, Mg-BGNs-1/DCECM, and Mg-BGNs-2/DCECM scaffolds in BMSC pellets using a Transwell system. The BMSC pellets were formulated as described in our previous study with 5 × 10^5^ BMSCs. The DCECM, Mg-BGNs-1/DCECM, and Mg-BGNs-2/DCECM scaffolds were transferred to the lower well of the Transwell plate (Corning Inc., Corning, NY, USA). The BMSCs were placed on the upper well of the Transwell plate (Corning, USA). Chondrogenic medium (Cyagen, Santa Clara, CA, USA) was added to the wells and changed every 3 days. After culturing in the well for 21 days, the pellets were harvested for analysis.

#### 2.3.2. Histological and Immunohistochemical Analysis

After being cultured in the Transwell for 21 days, the pellets were harvested and photographed. The pellets were fixed in 4% paraformaldehyde overnight, embedded in paraffin, and prepared into 7 μm sections. These sections were stained with hematoxylin-eosin (HE), Alcian blue (AB), and immunohistochemical (IHC) for collagen II (primary antibody Col II 1:100, Abcam, Boston, MA, USA).

#### 2.3.3. GAG/DNA Analysis

The GAG and DNA content of the pellets were determined according to our previous methods. We used the 1,9-dimethylmethylene blue (DMMB) assay method to measure GAG content using the Tissue GAG Total Content DMMB Colorimetry kit (Genmed Scientific Inc., Shanghai, China). DNA was extracted using the TIANamp Genomic DNA kit (TIANamp, Beijing, China) and quantified using the PicoGreen DNA assay kit (Invitrogen, Carlsbad, CA, USA).

#### 2.3.4. RT-PCR

The expression of chondrogenic-related genes (SOX9), collagen II, aggrecan, and collagen I) in the pellets treated with the DCECM, Mg-BGNs-1/DCECM, and Mg-BGNs-2/DCECM scaffolds were determined by real-time polymerase chain reaction (RT-PCR). The RT-PCR procedure was performed according to the general protocol. After extraction from the pellets with TRIzol (Invitrogen, Waltham, MA, USA), messenger ribonucleic acid (mRNA) was reverse transcribed into complementary DNA (cDNA) using a ReverTra Ace kit (Toyobo, Osaka, Japan), and then quantified using RT-PCR on a LightCycler 480 system (Roche Applied Science, Indianapolis, IN, USA). The primers used are listed in Table 1.

### 2.4. Statistical Analysis

Data are expressed as mean ± standard deviation (SD). Statistical analysis was performed using one-way analysis of variance (ANOVA) with SPSS Statistics version 22 (IBM, Armonk, NY, USA). A value of *p* < 0.05 was considered statistically significant.

## 3. Results

### 3.1. Characterization of Mg-BGNs

**Figure 1:**
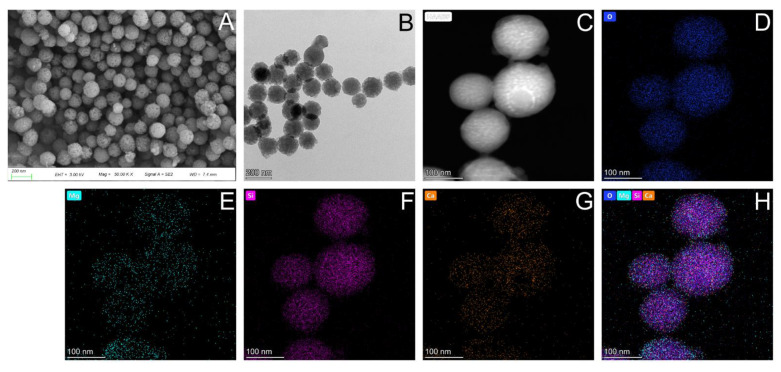
Characteristics of Mg-containing bioactive glass nanospheres (Mg-BGNs): (**A**) Scanning electron microscopy (SEM) image; (**B**) Transmission electron microscopy (TEM) image; (**C**) Scanning transmission electron microscopy (STEM) image; and (**D**–**H**) element maps (O, Mg, Si, Ca) of Mg-BGNs.

The nanoscale morphology of the Mg-BGNs was observed using field-emission scanning electron microscopy (FESEM) (Figure 1A) and field-emission transmission electron microscopy (FETEM) (Figure 1B). The average diameter of the uniformly sized nanospheres was 108.2 ± 22.5 nm. The chemical composition of the Mg-BGNs was characterized by scanning transmission electron microscopy (STEM)-energy dispersive spectroscopy (EDS). As shown in Figure 1C–H, elemental maps revealed that Mg, Si, Ca, and O are homogeneously distributed within the Mg-BGNs.

### 3.2. Morphology of DCECM and Mg-BGNs/DCECM Scaffolds

**Figure 2:**
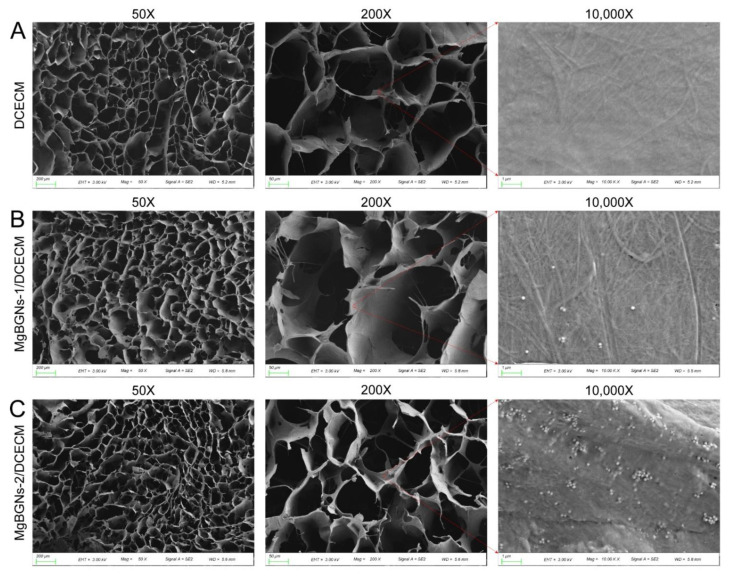
The microstructure of decellularized cartilage extracellular matrix (DCECM) and Mg-BGNs/DCECM scaffolds: (**A**) SEM images of DCECM scaffold; (**B**) SEM images of Mg-BGNs-1/DCECM scaffold; (**C**) SEM images of Mg-BGNs-2/DCECM scaffold.

The microstructures of the DCECM, Mg-BGNs-1/DCECM, and Mg-BGNs-2/DCECM scaffolds are shown in Figure 2. The SEM images clearly show that all three scaffolds exhibit a similar highly interconnected porous structure with a mean pore size of 80–200 μm. The high magnification images indicate that the Mg-BGNs-1/DCECM scaffold contains a small number of Mg-BGNs, whereas the Mg-BGNs-2/DCECM scaffold consists of abundant, uniform, and dispersed Mg-BGNs. The SEM results confirm that the Mg-BGNs tend to aggregate in the walls of the pores in the scaffolds.

### 3.3. Physicochemical Characterization of DCECM and Mg-BGNs/DCECM Scaffolds

**Figure 3:**
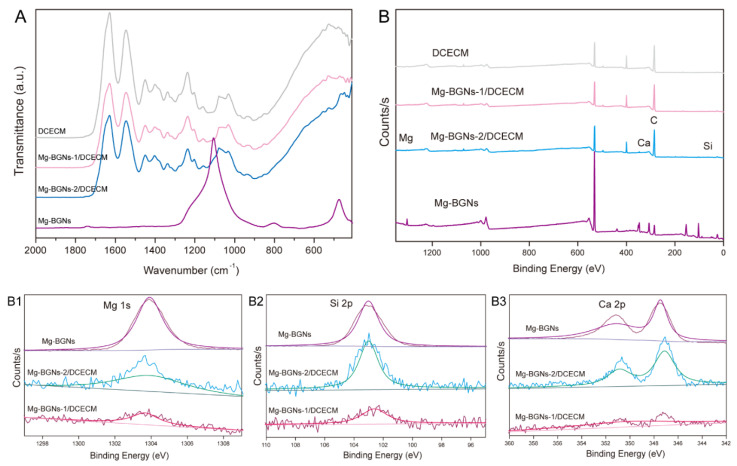
Physicochemical characterizations of Mg-BGNs/DCECM scaffolds: (**A**) Fourier transform infrared spectroscopy (FTIR) spectra of DCECM, Mg-BGNs-1/DCECM, Mg-BGNs-2/DCECM scaffolds, and Mg-BGNs; (**B**) The wide survey X-ray photoelectron spectroscopy (XPS) spectrum of DCECM, Mg-BGNs-1/DCECM, Mg-BGNs-2/DCECM, Mg-BGNs. The Mg 1s core-level spectra of Mg-BGNs-1/DCECM, Mg-BGNs-2/DCECM and Mg-BGNs is shown in (**B1**), the Si 2p core-level spectra is shown in (**B2**), and the Ca 2p core-level spectra is shown in (**B3**).

FTIR was used to evaluate the formation of Mg-BGNs and DCECM compounds. The FTIR spectra show a similar pattern for the DCECM, Mg-BGNs-1/DCECM, and Mg-BGNs-2/DCECM scaffolds. However, some additional bands and stretching vibrations at approximately 460 and 1030 cm^−1^ are observed in the Mg-BGNs-1/DCECM and Mg-BGNs-2/DCECM samples (Figure 3A). These were assigned to Si-O-Si, consistent with the silica network existing in the Mg-BGNs.

The wide-scan XPS analysis also shows that Mg, Ca, and Si atoms are present in the Mg-BGNs-1/DCECM and Mg-BGNs-2/DCECM samples (Figure 3B), which contains Mg-BG nanoparticles. In addition, the narrow scan of Mg 1s (Figure 3B1), Si 2p (Figure 3B2), and Ca 2p (Figure 3B3) shows a shift in binding energy in the Mg-BGNs, Mg-BGNs-1/DCECM, and Mg-BGNs-2/DCECM samples. This further confirms that Mg-BGNs are present in the Mg-BGNs-1/DCECM and Mg-BGNs-2/DCECM samples.

### 3.4. Ionic Release of Mg-BGNs/DCECM Scaffolds

**Figure 4:**
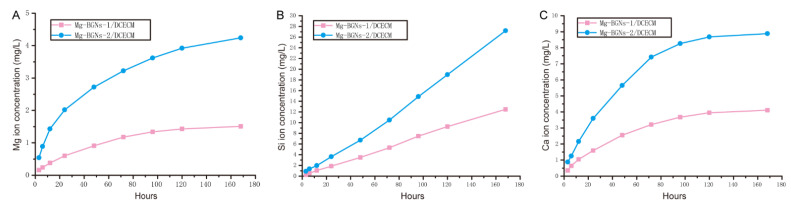
Amount of Mg (**A**), Si (**B**) and Ca (**C**) ions released from Mg-BGNs-1/DCECM and Mg-BGNs-2/DCECM scaffolds as a function of the soaking time in phosphate buffered saline (PBS).

The ionic release behaviors of the Mg-BGNs-1/DCECM and Mg-BGNs-2/DCECM scaffolds were examined using ICP-AES. The ionic concentrations of Mg, Si, and Ca in the scaffold extracts were recorded for up to 7 days, as shown in Figure 4. The release rate was rather rapid at first (up to 3 days) for Mg ions. This decreased slightly by day 7 (Figure 4A). The release rate of Si ions was relatively stable for up to 7 days (Figure 4B). The release behavior of Ca ions was similar to that of Mg ions—rapid up to 3 days and then decreasing slightly by day 7(Figure 4C). Overall, the Mg, Si, and Ca ionic release rates of the Mg-BGNs-2/DCECM scaffold are more rapid than those of the Mg-BGNs-1/DCECM scaffold.

### 3.5. Cell Viability Analysis of Scaffolds

**Figure 5:**
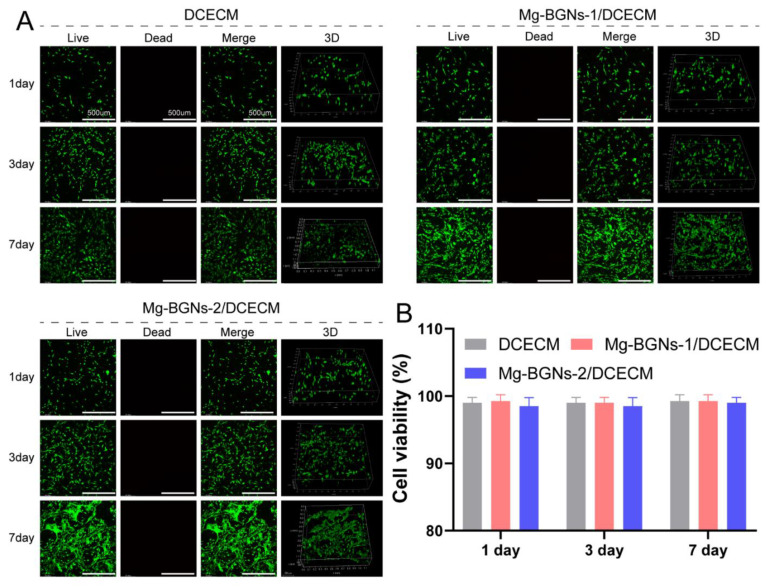
The cell viability analysis of the DCECM, Mg-BGNs-1/DCECM and Mg-BGNs-2/DCECM scaffolds: (**A**) The live/dead staining of bone marrow mesenchymal stem cells (BMSCs) seeded on scaffolds for 1, 3, and 7 days; representative images show live (green) cells, dead (red) cells, and 3D reconstruction images; (**B**) The viability analysis of BMSCs seeded on the DCECM, Mg-BGNs-1/DCECM and Mg-BGNs-2/DCECM scaffolds for 1, 3, and 7 days. Data are expressed as means ± standard deviation.

The cytocompatibility of the DCECM, Mg-BGNs-1/DCECM, and Mg-BGNs-2/DCECM scaffolds was evaluated using a live/dead cell staining method. BMSCs were seeded on the three different scaffolds and cultured for 1, 3, and 7 days. Subsequently, live/dead staining was performed and observed using a confocal microscope. The results are shown in Figure 5A. From this, we know that most of the cells in the scaffolds were live (green) and few cells died (red); this indicates that all the three different scaffolds had no obvious cytotoxicity. Quantitative cell viability analysis also confirms that the viability of BMSCs in DCECM, Mg-BGNs-1/DCECM, and Mg-BGNs-2/DCECM scaffolds after 1, 3, and 7 days was more than 95%, and there was no significant difference among the three different groups (Figure 5B).

### 3.6. Cell Proliferation and Attachment

**Figure 6:**
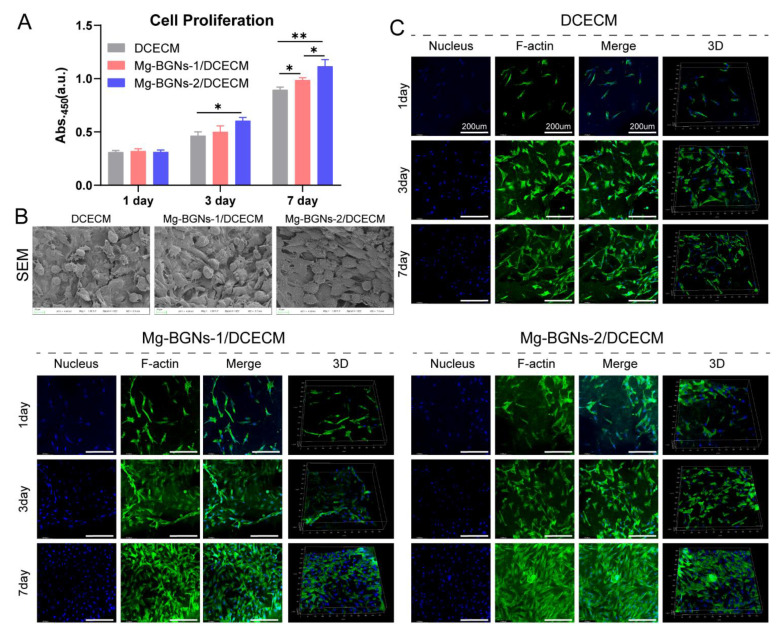
The cell proliferation, morphology and cytoskeletal architecture assessment of cells seeded on DCECM, Mg-BGNs-1/DCECM and Mg-BGNs-2/DCECM scaffolds: (**A**) The BMSCs proliferation analysis, which were seeded on DCECM, Mg-BGNs-1/DCECM and Mg-BGNs-2/DCECM scaffolds for 1, 3, and 7 days respectively; (**B**) The SEM images of BMSCs seeded on DCECM, Mg-BGNs-1/DCECM and Mg-BGNs-2/DCECM scaffolds for 3 days; (**C**) Confocal micrographs of the cytoskeletal architecture of BMSCs seeded on DCECM, Mg-BGNs-1/DCECM and Mg-BGNs-2/DCECM scaffolds for 1, 3, and 7 days. Data are expressed as mean ± standard deviation, * *p* < 0.05, ** *p* < 0.01.

The cell proliferation effect of the DCECM, Mg-BGNs-1/DCECM, and Mg-BGNs-2/DCECM scaffolds were evaluated using a CCK-8 assay kit. As shown in Figure 6A, on day 1, there was no significant difference between the three different groups. However, the cell proliferation effect of the Mg-BGNs-2/DCECM group was higher than the DCECM group (*p* < 0.05) on day 3. On day 7, the Mg-BGNs-2/DCECM group’s cell proliferation effect was higher than the Mg-BGNs-1/DCECM group, and this, in turn, was higher than the DCECM group (*p* < 0.05). The results indicate that Mg-BGNs-2/DCECM scaffolds can promote the proliferation of BMSCs compared to the Mg-BGNs-1/DCECM and DCECM groups. The BMSCs attached to the DCECM, Mg-BGNs-1/DCECM, and Mg-BGNs-2/DCECM scaffolds were observed using SEM (Figure 6B). It can be seen that BMSCs spread well on the DCECM, Mg-BGNs-1/DCECM, and Mg-BGNs-2/DCECM scaffolds. Cell attachment and growth were also observed by F-actin staining. As shown in Figure 6C, the cytoskeletal morphology of BMSCs seeded on the DCECM, Mg-BGNs-1/DCECM, and Mg-BGNs-2/DCECM scaffolds showed that all the BMSCs on the three scaffolds appeared to grow and spread well after culturing for 1, 3, and 7 days. They also exhibited elongated, multilayered morphologies with the extended spreading of actin filaments.

### 3.7. Chondrogenic Differentiation Induction of Scaffolds

**Figure 7:**
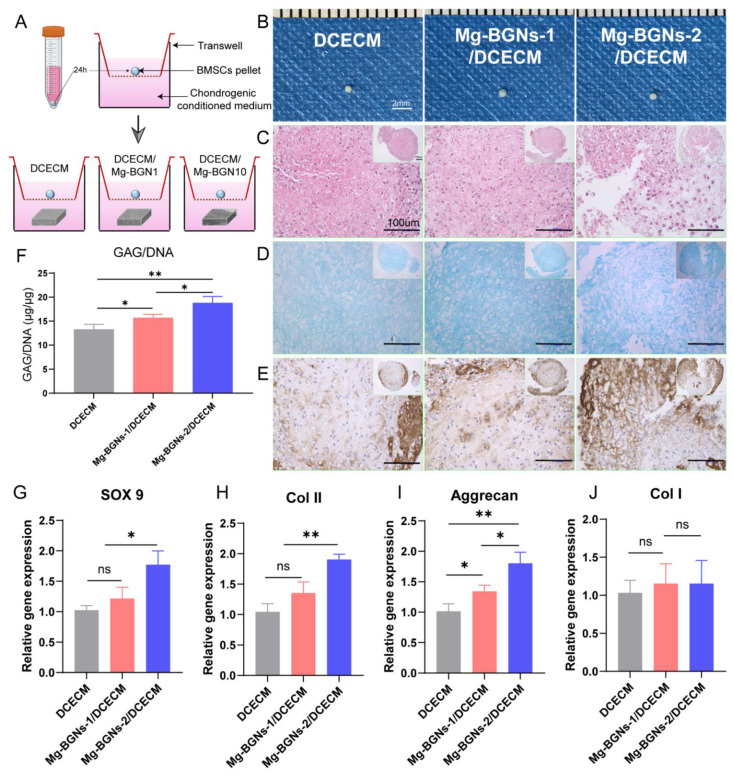
The chondrogenic effects of the DCECM, Mg-BGNs-1/DCECM and Mg-BGNs-2/DCECM scaffolds: (**A**) General scheme of the in vitro induction of chondrogenesis in BMSC pellets treated with DCECM, Mg-BGNs-1/DCECM and Mg-BGNs-2/DCECM scaffolds; (**B**) The gross observation of the pellets treated with DCECM, Mg-BGNs-1/DCECM and Mg-BGNs-2/DCECM scaffolds for 21 days; (**C**) HE staining of the pellets; (**D**) AB staining of the pellets; (**E**) collagen II staining of the pellets; (**F**) GAG normalized by DNA content of the pellets; data are expressed as mean ± standard deviation (*n* = 3; * *p* < 0.05, ** *p* < 0.01). (**G**–**J**), RT-PCR of SOX 9 (**G**); Collagen II (**H**); aggrecan (**I**); and Collagen I (**J**) from BMSC pellets treated with DCECM, Mg-BGNs-1/DCECM and Mg-BGNs-2/DCECM scaffolds for 21 days. Data are expressed as mean ± standard deviation (*n* = 3; * *p* < 0.05, ** *p* < 0.01).

We evaluated the chondrogenic differentiation induction capacity of the DCECM, Mg-BGNs-1/DCECM, and Mg-BGNs-2/DCECM scaffolds in BMSC pellet culture in vitro. The pellets were cultured in a chondrogenic conditioned medium for 21 days, as shown in Figure 7B. Figure 7C–E shows the histological observation of the pellets for HE, AB, and ICH of collagen II staining. The AB staining shows the greatest intensity in the pellets treated with Mg-BGNs-2/DCECM. The IHC collagen II of the Mg-BGNs-2/DCECM group was greater than that of the Mg-BGNs-1/DCECM group, which was also greater than that of the DCECM group.

To evaluate the glycosaminoglycan (GAG) content of the pellets treated with the DCECM, Mg-BGNs-1/DCECM, and Mg-BGNs-2/DCECM scaffolds, we calculated GAG normalized for DNA content. GAG per DNA content of the Mg-BGNs-2/DCECM group was significantly higher than that of the Mg-BGNs-1/DCECM group (*p* < 0.05). This was also higher than that of the DCECM group (*p* < 0.05) (Figure 7F).

The expression of chondrogenic differentiation-related genes (SOX 9, Col II, Aggrecan, and Col I) in the pellets treated with the DCECM, Mg-BGNs-1/DCECM, and Mg-BGNs-2/DCECM scaffolds were evaluated by RT-PCR. The SOX 9 expression of the pellet treated with Mg-BGNs-2/DCECM is higher than that of the DCECM and Mg-BGNs-1/DCECM group (*p* < 0.05) (Figure 7G). The Col II expression of the Mg-BGNs-2/DCECM group is higher than that of the DCECM and Mg-BGNs-1/DCECM groups (*p* < 0.01) (Figure 7H). The aggrecan expression of the Mg-BGNs-2/DCECM group is higher than that of the Mg-BGNs-1/DCECM group, which is, in turn, higher than that of the DCECM group (*p* < 0.05) (Figure 7I). There are no significant differences in the Col I expressions among the three groups (Figure 7J). All the results indicate that the Mg-BGNs-2/DCECM has a better chondrogenic differentiation induction effect than DCECM and Mg-BGNs-1/DCECM.

## 4. Discussion

Osteochondral tissues are the continuous organization that involves both the articular cartilage and subchondral bone tissue, and both tissues play a significant role in OA onset and progression. The ideal treatment strategy not only promotes cartilage repair but also enhances subchondral bone regeneration. Tissue engineering scaffolds with the capacity to simultaneously stimulate cartilage and subchondral bone regeneration may be an ideal therapeutic strategy for osteochondral lesions. Bioactive glasses are promising biomaterials for the treatment of osteochondral injuries due to their dual functions in bone and cartilage regeneration. A number of studies have already confirmed that bioactive glasses can promote osteogenic differentiation of MSCs and are beneficial for bone tissue regeneration [33,34]. In recent years, some studies have found that some therapeutic ions, such as Mg and Si, have some beneficial effects on cartilage regeneration. Mg plays a key role in cellular energy metabolism and has a positive effect on chondrogenesis of MSCs through integrin-signaling proteins, TGFB1, octamer-binding protein (NONO), and others [35]. Furthermore, Mg can enhance chondrocyte growth and cartilage extracellular matrix synthesis (aggrecan and collagen II) [20]. Si can stimulate cartilage reconstruction by activating the HIF pathway and has a positive effect on OA therapy [36]. Considering that bioactive glasses are excellent carrier systems for ions, bioactive glasses containing therapeutic ions might be ideal tissue engineering biomaterials for osteochondral regeneration.

In this study, we fabricated sol-gel-derived Mg-BGNs. We then incorporated Mg-BGNs into a DCECM to construct a Mg-BGNs/DCECM composite scaffold. The overall aim of the present study was to investigate the effects of incorporating Mg-BGNs into DCECM scaffolds. These include the microarchitectural and physicochemical properties, cytocompatibility, and the ability of the scaffolds to enhance chondrogenic differentiation in BMSC pellet culture. The results demonstrate that Mg-containing bioactive glass nanospheres can be successfully fabricated by the sol-gel method. Furthermore, the three scaffolds (DCECM, Mg-BGNs-1/DCECM, and Mg-BGNs-2/DCECM scaffolds) constructed via the freeze-drying method were highly porous, had suitable pore size for tissue engineering, and sustainably released Mg, Si, and Ca ions. Moreover, all three scaffolds have excellent biocompatibility, showing no signs of toxicity. Most importantly, the addition of Mg-BGNs not only resulted in cell proliferation but also enhanced chondrogenic differentiation of MSCs in the pellet culture in a dose-dependent manner. Collectively, these results indicate that the Mg-BGNs/DCECM scaffold developed in this study shows potential for use in osteochondral lesion repair.

We proposed the development of a multifunctional composite scaffold for the treatment of osteochondral lesions, which requires a matrix that is not only capable of supporting cartilage regeneration but is also capable of contributing to bone regeneration. Bioactive glass is an ideal biomaterial for bone regeneration. Various studies have already explored its osteogenic capabilities. Therefore, in this study, we focused on the induction of chondrogenic differentiation by Mg-BGNs. We found that the incorporation of Mg-BGNs into the DCECM scaffold successfully enhances the chondrogenic differentiation of MSCs in pellet culture. The Mg-BGNs-2/DCECM scaffold resulted in a significant increase in GAG secretion in comparison to the Mg-BGNs-1/DCECM, which also had a higher GAG content than the DCECM control group. The AB and Col II staining indicates that the pellet treated with Mg-BGNs-2/DCECM shows higher intensity than that of Mg-BGNs-1/DCECM and DCECM. Additionally, the RT-PCR results also show that the expression of the chondrogenic differentiation-related genes SOX 9, Col II, and Aggrecan in the Mg-BGNs-2/DCECM group are higher than those of the DCECM control group.

To the best of our knowledge, this is the first study to successfully demonstrate the incorporation of Mg-containing bioactive glass nanospheres into a natural DCECM scaffold. Before freeze-drying, we added Mg-BGNs to the DCECM hydrogels and made them homogeneously distributed throughout the hydrogels. We fabricated three different scaffolds: DCECM, Mg-BGNs-1/DCECM, and Mg-BGNs-2/DCECM. The SEM results indicate that the pore sizes of all three scaffolds are similar and within the range of 80–200 μm, which is the ideal range for tissue engineering applications according to a previous study [37]. The FTIR and XPS results confirm the successful incorporation of Mg-BGNs into the DCECM scaffold. Additionally, ICP-AES analysis demonstrates that Mg-BGNs/DCECM can control the release of Mg, Si, and Ca ions in a dose-dependent manner. Cell viability analysis indicates that all three scaffolds show excellent cytocompatibility and no signs of toxicity. Cytocompatibility analysis shows that all three scaffolds promote cell adhesion and growth. The cell proliferation analysis indicates that there is a significant increase in cell number on the scaffolds containing Mg-BGNs, which may be attributed to the ionic extracts from the Mg-BGNs/DCECM scaffold. In addition, it has been found that the combination of Mg-BGNs with DCECM could significantly promote chondrogenesis in comparison to the DCECM-only control. Thus, we can postulate that the enhanced cartilage regenerative capacity of the Mg-BGNs/DCECM scaffold can be attributed to the combination of Mg-containing bioactive glass nanospheres.

This study had some limitations. First, it only focused on the assessment of the feasibility of the application of Mg-BGNs/DMECM composite scaffolds for cartilage regeneration in vitro. The assessment of these novel materials in an in vivo study will be performed in our next study. Second, it must be highlighted that the incorporation of bioactive glass into DCECM scaffolds would alter the stiffness of the construction, and this may also influence the differentiation of MSCs. As this study only focused on the in vitro assessment, the biomechanical variation of the addition of BG into DCECM scaffolds would also be performed in our next study, together with the in vivo study. Third, the containing certain therapeutic ions (like magnesium) bioactive glass could favor hydroxyapatite (HA) formation, because magnesium and calcium could be incorporated into hydroxyapatite crystals, while HA deposition is unwanted in cartilage. However, it was unclear whether the containing certain therapeutic ions bioactive glass could cause heterotopic ossification in the cartilage layer. This is also a limitation of this study, and we will further investigate the HA deposition issue possibly produced by the bioactive glass in the following study. Furthermore, the composite scaffold used in this study was homogeneous architecture, while the osteochondral structure was heterogeneous. Finally, although many studies have confirmed that bioactive glasses are widely used for bone tissue regeneration, the osteogenic capacity of the Mg-BGNs/DCECM composite scaffold is still not very clear. We would like to evaluate its effect on osteogenesis in a future study.

## 5. Conclusions

In this study, Mg-containing bioactive glass nanospheres (Mg-BGNs) were successfully prepared using the sol-gel method and incorporated into the DCECM to construct a Mg-BGNs/DCECM composite scaffold. The Mg-BGNs/DCECM composite scaffold had a highly porous structure, suitable pore size for tissue engineering, good biocompatibility, promoted MSC proliferation and showed enhanced chondrogenic differentiation in comparison to the DCECM scaffold. Given the results of this study, we suggest that the Mg-BGNs/DCECM composite scaffold can simultaneously stimulate articular cartilage and subchondral bone regeneration, which satisfies the requirement for osteochondral lesions, and provides an alternative selection for cartilage/bone regeneration.

## Figures and Tables

**Table 1:** The Primer used in this study.

Gene	Primer
SOX 9	Forward: GCGGAGGAAGTCGGTGAAGAAT
	Reverse: AAGATGGCGTTGGGCGAGAT
Col II	Forward: CACGCTCAAGTCCCTCAACA
	Reverse: TCTATCCAGTAGTCACCGCTCT
Aggrecan	Forward: GGAGGAGCAGGAGTTTGTCAA
	Reverse: TGTCCATCCGACCAGCGAAA
Col I	Forward: GCCACCTGCCAGTCTTTACA
	Reverse CCATCATCACCATCTCTGCCT
GAPDH	Forward: CAAGAAGGTGGTGAAGCAGG
	Reverse: CACTGTTGAAGTCGCAGGAG

## Data Availability

Not applicable.
